# Comprehensive collection of COVID-19 related prosthetic valve failure: a systematic review

**DOI:** 10.1007/s11239-022-02746-x

**Published:** 2022-12-17

**Authors:** Theresa K. Trieu, Kade Birkeland, Asher Kimchi, Ilan Kedan

**Affiliations:** 1 College of Medicine, California Northstate University, Elk Grove, CA USA; 2 Enterprise Data Intelligence, Cedars-Sinai Medical Center, Los Angeles, CA USA; 3 Smidt Heart Institute, Cedars-Sinai Hospital, 8501 Wilshire Blvd Suite 200, Beverly Hills, Los Angeles, CA USA

**Keywords:** Prosthetic valve thrombosis, Mechanical valve, Bioprosthetic valve, COVID, Thrombosis

## Abstract

Since the beginning of the SARS-CoV-2 (COVID-19) pandemic, correlation of venous thromboembolism (VTE) and COVID-19 infection has been well established. Increased inflammatory response in the setting of COVID-19 infection is associated with VTE and hypercoagulability. Venous and arterial thrombotic events in COVID-19 infection have been well documented; however, few cases have been reported involving cardiac valve prostheses. In this review, we present a total of eight cases involving COVID-19-related prosthetic valve thrombosis (PVT), as identified in a systematic review. These eight cases describe valve position (mitral versus aortic) and prosthesis type (bioprosthetic versus mechanical), and all cases demonstrate incidents of PVT associated with simultaneous or recent COVID-19 infection. None of these eight cases display obvious non-adherence to anticoagulation; five of the cases occurred greater than three years after the most recent valve replacement. Our review offers insights into PVT in COVID-19 infected patients including an indication for increased monitoring in the peri-infectious period. We explore valve thrombosis as a mechanism for prosthetic valve failure. We describe potential differences in antithrombotic strategies that may offer added antithrombotic protection during COVID-19 infection. With the growing population of valve replacement patients and recurring COVID-19 infection surges, it is imperative to explore relationships between COVID-19 and PVT.

## Highlights

- COVID-19 related prosthetic valve failure has been reported in prosthetic valve patients of varying valve prosthesis types.
- Prosthetic valve failure and valve thrombosis with COVID-19 infection has been reported in patients while on antithrombotic therapy.
- With known hypercoagulability associated with COVID-19 infection, prosthetic valve failure and valve thrombosis is an important diagnosis to consider in COVID-19 infected patients.
- Future study may better identify which patients may be at increased risk of prosthetic valve failure and valve thrombosis in the setting of COVID-19 infection.

## Introduction

Since the beginning of the Sars-CoV-2 (COVID-19) pandemic, the relationship between infection and hypercoagulability has been well documented [1]. It is hypothesized that COVID-19 infected patients develop a hypercoagulable state as a result of increased inflammatory cytokine production and upregulation of coagulation pathways increasing the risk of developing thrombotic events [2–4]. While many of these COVID-19-related thrombotic events such as deep vein thrombosis (DVT), pulmonary embolism (PE), cerebrovascular accident (CVA), and myocardial infarction (MI) have been documented and reviewed [4], few have been reported as prosthetic valve thrombosis (PVT). Our goal is to collect and summarize all published cases of PVT associated with COVID-19 infection.

## Methods

A systematic literature search in PubMed was conducting using the four search terms “COVID and Aortic Valve,” “COVID and Mitral Valve,” “COVID and Tricuspid Valve,” and “COVID and Pulmonic Valve” to identify PVT incidents in the setting of COVID-19 infection. Only articles that were published before July 14, 2022 were included. Each article was then reviewed and included if it was a case report deemed to be presenting PVT in the setting of COVID-19 infection. For each article, data on the following were gathered: the relation of time between valve replacement, COVID-19 infection, and PVT; the type (bioprosthetic vs mechanical) and position (mitral vs aortic) of the involved valve; prior anticoagulation/antiplatelet therapy; D-dimer levels; COVID-19 infection severity; history of other significant conditions; thrombus descriptions; imaging results; additional complications/consequences; treatments; and outcomes. Exclusion criteria included non-English publications, articles unrelated to prosthetic valve failure, articles about prosthetic valve failure outside the context of COVID-19 infection, and articles accounting COVID-19-related prosthetic valve endocarditis. Data was compiled using Excel.

## Results

**Fig. 1:**
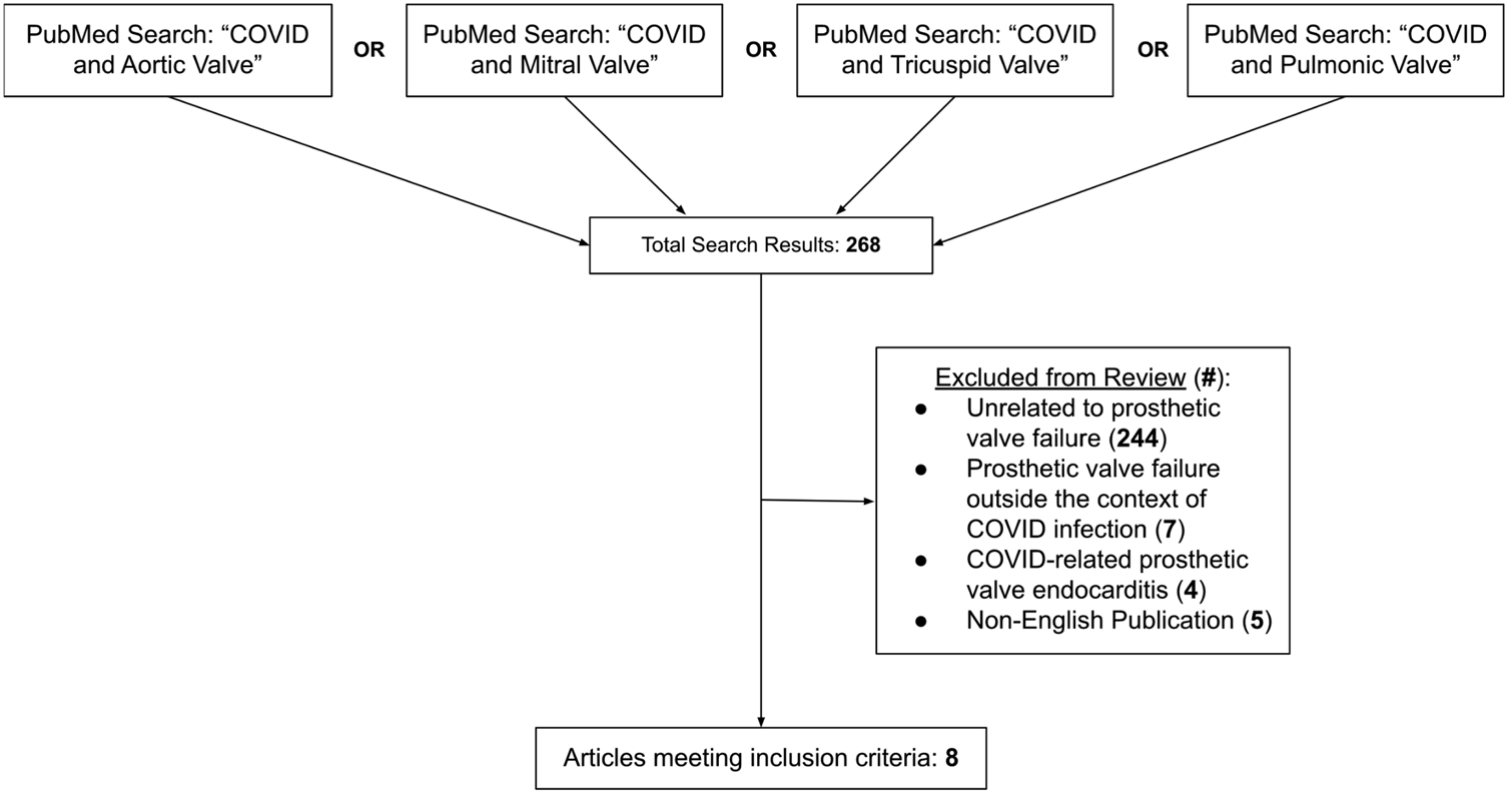
Results from systematic PubMed literature review using search terms: “COVID and aortic valve”, “COVID and mitral valve”, “COVID and tricuspid valve”, and “COVID and pulmonic valve”

The initial literature review resulted in 268 journal articles. Of these, 244 were deemed unrelated to the focus of prosthetic valve failure, seven documented prosthetic valve failure outside the context of recent/simultaneous COVID-19 infection, and another four recorded COVID-19-related prosthetic valve endocarditis. Five were non-English publications. The remaining eight articles were all case studies and documented COVID-19-associated prosthetic valve thrombosis, the objective of this review (Fig. 1).

The eight COVID-19-related PVT cases documented in this paper include valve type (mitral versus aortic) and prosthesis type (bioprosthetic versus mechanical). Of these cases, five involved prostheses in the mitral valve position, three of which were mechanical [5–7] and two were bioprosthetic [8, 9]; three of the eight cases involved aortic valve prostheses, one being mechanical [10] and two being surgical bioprosthetic valves [11, 12] (Table 1). Seven of eight patients had symptoms of dyspnea or hypoxia [5–9, 11, 12]. Al Helali et al. presented a patient with a mechanical aortic valve prosthesis presenting with a change in character of cardiac auscultation and generalized fatigue [10].

**Table 1:** Comprehensive summary of COVID-related PVT cases [5–12]

Author(s)	Paper	Valve	Prosthesis type	Age/gender	Other complications	Replacement indication	Replacement & COVID timeline	Prior medications	Labs
Jeckelmann et al	Case report: Mechanical mitral prosthetic valve thrombosis in the context of COVID-19 despite effective anticoagulation	Mitral	Mechanical (Medical Medtronic ATS 27 mm)	58 yo female	New AF related to thrombosis of mitral mechanical valve, COVID pneumonia, indirect signs of pulm htn, & respiratory failure	Severe mitral stenosis	- Replacement 6 yrs prior. Admitted with COVID resp failure (having tested pos 2 days prior)	For mechanical valve anticoag, acenocoumarol tx, INR mostly in therapeutic range 2.5–3.5. Also, 6 days before admission, 10 mg oral vit K following INR 7.7	Electrolytes in normal range, normal renal and liver function. Troponin T hs was 16 ng/L (n < 14 ng/L), N-terminal prohormone of brain natriuretic peptide 3146 ng/L (n < 300 ng/L), D-dimers 6893 mcg/L (n < 350 mcg/L), INR 2.7
Cardona Buitrago et al	Mechanical valve thrombosis secondary to severe acute respiratory syndrome coronavirus 2 infection: a Case report	Mitral	Mechanical (St. Jude Medical 29 mm; St. Paul, MN: St. Jude Medical, Inc.)	52 yo female	- Other history: permanent valvular AF, obesity, congenital single kidney (glomerular filtration rate {GFR}: 89.9 mL/min), and hypothyroidism - Pneumonia - During recovery after current hospitalization & replacement: bradyarrhythmia w rhythm of excape from the junction, PVCs of 2 mrophologies, piars, bigeminy, episodes of NSVT	Rheumatic fever	- Replacement 8 yrs prior. Confirmed 20 days ago with COVID. Admitted now with respiratory difficulty & generalized edematous syndrome	Warfarin	Not mentioned
Aruğaslan et al	Mechanical Mitral Valve Thrombosis in a Patient with COVID-19 Infection	Mitral	Mechanical	46 yo male	- Atypical COVID-19 (bilateral centrilobular infiltrations) - on day 7: SVT & pulm edema	Not mentioned	- Replacement 3 yrs prior, admitted with 1 week h/o mild dyspnea & malaise. Tested pos for COVID during hospital course	Warfarin 5 mg/day	- Admission INR was 3.26. mild hypoxemia - D dimer 1.0 mg/L (< 0.55), C- reactive protein 0.02708 g/L (0–- 0.005), IL-6 14.7 pg/mL (0 – 3.4), platelets 258 × 10^9/L(150–400), and ferritin 58 µg/L (22 – 322)
Llopis Gisbert et al	Bioprosthetic Valve Thrombosis and Obstruction Secondary to COVID-19	Mitral	Bioprosthetic	77 yo male	- Simultaneous PE - Pulm congestion w bilateral pleural effusion	Staph aureus endocarditis w severe valve regurg	Replacement 2017, 2020 admitted for COVID pneumonia & discharged, 2 weeks afterwards presented w PE & severe prosthetic mitral stenosis	Not mentioned	During hospitalization 2 weeks later, normal platelet count & high D-dimer. (3041 ng/mL) (see supplemental table https://www.ncbi.nlm.nih.gov/pmc/articles/PMC7585166/bin/mmc1.docx)
Vinnakota et al	Thrombolysis for COVID-19-associated bioprosthetic mitral valve thrombosis with shock	Mitral	Bioprosthetic (23 mm Edwards Sapien 3 Ultra)	63 yo female	other history: end-stage renal failure on haemodialysis, AF on apixaban	- First replacement (aortic & mitral): indication not mentioned - Second replacement (mitral valve-in-valve implant): severe early mitral bioprosthetic stenosis, presumed secondary to bioprosthetic thrombosis	- Timeline: mitral & aortic valve replacements, 12 mo later undergoes transcatheter mitral valve-in-valve implantation, 7 mo afterwards diagnosed w COVID-19, 1 mo afterwards presents to hospital (unresponsive, hypotensive, hypoxic)	Apixaban (2.5 mg BID; for AF) due to prior warfarin-associated bleeding from calciphylaxis wounds	Not mentioned
Alexander et al	Bioprosthetic valve thrombosis associated With COVID-19 infection	Aortic	Bioprosthetic	79 yo female	COVID pneumonia 2 mo prior	Type A aortic dissection w hemiaortic arch	Underwent AVR, 2 mo after was hospitalized for COVID pneumonia, then 2 mo after presented w dyspnea/cough and found prosthetic valve stenosis	After COVID hospitalization, was discharged on apixaban 5 mg twice daily	- First admission (for COVID pneumonia): elevated C-reactive protein (79.8 mg/dL), activated partial thromboplastin time (44.2 s), fibrinogen (489 mg/dL), and D-dimer (2.14 μg/mL)
Manghat et al	Acute postoperative thrombosis of an aortic valve prosthesis and embolic myocardial infarction in a coronavirus disease 2019 (COVID-19)-positive patient-an unrecognized complication	Aortic	Bioprosthetic (25-mm diameter Carpentier-Edwards PERIMOUNT (Edwards Lifesciences, Irvine, Calif)	73 yo female	Embolic MI (EKG & troponin-T findings), PE, pericardial effusion; transient atrial flutter, symptoms of retinal artery embolism	Critical bicuspid valve stenosis. Also received left internal mammary artery graft to the left anterior descending artery (LAD)	14 days postop, readmitted. ^2n^d COVID-19 swab was positive	75 mg aspirin	Findings of the coagulopathy panel were normal prothrombin time (10.9 s), elevated platelets (488 × 109/L), fibrinogen (6.5 g/L), and D-dimers (5547 ng/mL)
Al Helali et al	Successful use of ultraslow thrombolytic therapy in stuck mechanical aortic valve in a patient with COVID-19; a case report	Aortic	Mechanical (AV, St Jude size 21 mm)	37 yo male	Not mentioned	Severe rheumatic aortic regurgitation, with baseline transvalvular mean gradient of 40 mm Hg	- Replacement 1998. Tested positive for COVID-19 on this admission	Aspirin & warfarin	Platelet count of 317 1000/μL, prothrombin time (PT) of 15 s, activated partial thromboplastin time (aPTT) of 36 s, international normalized ratio (INR) of 1.1, C-reactive protein (CRP) of 7.0 mg/L, ferritin of 646 ng/mL, and D-dimer of 372 ng/mL

Author(s)	Management	Discharge medications	TTE after initial replacement	TTE	Fluorography	Stress Perfusion MRI	TEE	CT	F/U Studies
Jeckelmann et al	Mechanical ventilation, stopped VKA tx, started enoxaparin (80 mg 2 × /day SQ). After suspecting valve thrombosis, IV heparin given. No improvement in 1 week (fluorography performed), so low dose IV thrombolysis alteplase (rtPA) given (10 mg bolus followed by 1.5 mg/kg IV infusion over 2 h). Fluorography next day & TTE showed some improvement. Next day VKA treatment with phenprocoumon was initiated and heparin was continued until therapeutic INR was achieved. REG AF: rate control strategy was chosen for AF management using beta-blockers and digoxin.Clinical course favorable, pt discharged a week later	Not mentioned	Not mentioned	Reduced mobility of the two leaflets of the mechanical mitral prosthesis with an image suggestive of thrombus. Transprothetic mitral Doppler peak velocity was elevated (254 cm/s), pressure half time was prolonged (213 ms). The mean transprosthetic gradient (TPG) was 16 mmHg. The LA was severely dilated (90 mL–59 mL/m2). Pulmonary hypertension was likely (tricuspid insufficiency speed 341 cm/s and interventricular septal D-shaping) with an estimated pulmonary arterial pressure (PAP) of 57/32-39 (s/d-mean) mmHg. Left ventricular ejection fraction was 55%.	Complete immobility of one of the leaflets and hypo-mobility of the other	n/a	BRIEF: 9 mm × 6 mm hypoechogenic mass attached to one of the leaflets. DETAILED: (1) a dilated left atrium with; (2) an isoechoic mass straddling the leaflet hinge (3) posterior leaflet and (4) anterior leaflet	Thoracic: typical COVID lung lesions, & massively dilated LA	1 day after alteplase, fluorography showed normalization of mobility of one of the valve leaflets, the other remained immobile. F/U TTE showed decrease in TPG of 4 mmHg and a functional mitral valve area of 1.8 cm2, pulmonary arterial pressure was estimated at 39/8–18 (s/d-m) mmHg. 1 YR F/U TTE showed TPG of 4 mmHg, mitral valve area of 4.25 cm2, and PAP estimated at 26 mmHg. Asymptomatic permanent AF at 72 b.p.m. was diagnosed
Cardona Buitrago et al	- Administered warfarin - non-invasive mechanical ventilation & parenteral steroid (clinical improvement) - bc of hypoperfusion & low CO & prosthesis thrombus: double vasopressor, and emergency valve replacement surgery (St. Jude No. 29; St. Paul, MN: St. Jude Medical, Inc.) - while in ICU for recovery, experienced some PVCs and NSVT, so given beta-blockers and antiarrhythmics. Also received unicameral pacemaker - The clinical course was favorable, and the patient was discharged when he reached the therapeutic INR (INR at hospital discharge was 3.57 s)	not mentioned	not mentioned	A hyperechogenic, mobile image of 7 x 9 mm was shown at the lateral level of the mitral prosthesis toward the ventricular side, one of the discs showed excursion without excursion of the second disc. See TEE for better characterization	n/a	n/a	- Thrombus (20 × 15 × 20 mm) in the lateral disc of the mechanical prosthesis, restricting its mobility. maximum gradient of 39, mean gradient of 22 mmHg, effective hole area estimated by pressure half time (THP) of 1.2 cm2	- Chest: organized pneumonia in the right apical lobe	- 2 mo f/u transthoracic US: normofunctional mitral prosthesis demonstrated, without evidence of thrombi or intracavitary masses
Aruğaslan et al	- Presented with dyspnea and malaise, absence of prosthetic click - warfarin stopped, IV unfractionated heparin given - on day 7, upon SVT & pulm edema & re-elevation of mean pressure gradient to 28: emergent thrombolytic (10 mg bolus of tPA and 90 mg infusion in 90 min). No amelioration seen - urgent mitral valve replacement done, thrombosed mechanical valve observed & excised. Replaced with new mechanical valve (29 mm, Sorin) - discharged with target INR 3.5	not mentioned	not mentioned	- Initial TTE: severely restricted leaflet mobility, with a mean transvalvular gradient of 23 mmHg (Figure 1). Obstructive thrombus with a 2.2 X 0.8 cm diameter extending to the left ventricular outflow tract was seen- on ^3r^d day of tx: decreased mitral valve gradients (mean 12 mmHg)- bedside echo on day 7: re-elevation of the mean pressure gradient to 28 mmHg.	Restricted mobility of leaflets	n/a	n/a	- Chest: bilateral centrilobular infiltrations	Not mentioned
Llopis Gisbert et al	- During covid hospitalization, received anticoagulation at prophylactic doses (3500 IU bemiparin every 24 h) - During hospitalization 2 weeks later, standard treatment for heart failure with intravenous diuretics and weight-adjusted anticoagulation with low-molecular-weight heparin as part of the treatment for PE	Vitamin K antagonist (acenocoumarol) for an international normalized ratio goal of 3-3.5, bridging with heparin. Reevaluate in 3mo	Nov 2019: normal prosthesis function (transmitral gradient of 4 mm Hg)	Severe prosthetic mitral stenosis; The mitral valve area estimated by pressure half-time was 0.5-0.6 cm2, and the mean transprosthetic gradient was 20 mm Hg, with pulmonary artery systolic pressure estimated to be 85 mm Hg and a dilated right ventricle with systolic dysfunction (TAPSE 11 mm, S′ 6.2 cm/s)	n/a	n/a	Mesoesophageal 2-chamber view showing thickened leaflets compatible with laminar thrombus with limited mitral valve opening (valve orifice area of 0.6 cm2 as determined by means of 3-dimensional planimetry) and a high mean gradient (19 mm Hg)	n/a	15 days after last admission: TEE confirmed resolution of the prosthetic thrombus and stenosis with thin leaflets, normal valve opening (planimetry of 2.1 cm2), andnormalization of the transprosthetic mean gradient (5 mm Hg), and only mild anterior leaflet restriction persisting with complete telediastolic opening
Vinnakota et al	- After CT, administered weight-based IV tenecteplase emergently - long-term warfarin with anti-platelet therapy was initiated prior to discharge	Long-term warfarin with antiplatelet therapy	Not mentioned	n/a	n/a	n/a	- 30 min post-thrombolytics: large burden of mitral bioprosthetic thrombosis - 90 min post-thrombolytics: near complete resolution	Large thrombus within the mitral bioprosthesis, resulting in near total occlusion of inflow	- Repeat CT chest 2 days later: confirmed persistent resolution of thrombus
Alexander et al	- For COVID hospitalization: convalescent plasma; prophylactic LMWH, bridged to warfarin with a goal internationalized normalized ratio of 2.5 to 3.5 - for current hospitalization: started on unfractionated heparin infusion, and bridged to warfarin w goal INR of 2.5–3.5	Not mentioned	Not mentioned	Prosthetic aortic valve stenosis with a peak aortic velocity of 3.8 m/s, increased from 2.2 m/s postoperatively	n/a	n/a	Markedly thickened bioprosthetic valve leaflets	Hypoattenuated leaflet thickening involving all 3 valve leaflets consistent with leaflet thrombosis	3 mo later, TTE showed improvement of her aortic valve gradient back to baseline
Manghat et al	- Initial supportive oxygen and vasoconstrictor therapy resulted in clinical improvement - 4 days after admission: IV heparin & concomitant warfarin (discharged 6 days later)	not mentioned	not mentioned	Obesity limited clarity; pericardial effusion 1.9 cm in depth, no tamponade or prosthetic valve pathology with leaflets working well	n/a	Typical extensive acute LAD territory MI with microvascular obstruction but no ischemia, tamponade, or myocarditis. Susceptibility metallic artifact (Fig. 1, A) inhibited AVR assessment	n/a	- Agio: left lower-lobe PE, moderate-sized hemorrhagic pericardial effusion, patent left internal mammary artery to LAD graft, no acute aortic pathology - cardiac radiologist CT review: extensive low-attenuation, lobulated, and fragmented soft-tissue thrombus formation seen in the vicinity of and applied to the valve prosthesis frame (but not the leaflets) and in close proximity to the left main stem ostium, evidence of a small acute apical MI, probable evolving perioperative hemorrhagic pericardial effusion but no tamponade or contrast extravasation and no left atrial or appendage thrombus	- repeat CT 11 weeks post anticoags: complete resolution of AVR thrombosis
Al Helali et al	- At regular f/u visit, presented w generalized fatigue & loss of one of the mechanical heart sounds for 10 days - tests pos for COVID - ultraslow thrombolytic therapy: alteplase 1 mg, every hour for 25 h, followed by 6 h of unfractionated heparin (70 u/kg bolus then 16 u/kg/h with a target aPTT of 1.5 to 2.0 times the control value) - discharged after 7 days with INR 3.0 for 2 consec days & continued aspirin	not mentioned	not mentioned	- Initial: high peak and mean AV gradients (170 and 80 mm Hg, respectively) and moderate intrinsic aortic regurgitation (AR). Additionally, there were moderate mitral and tricuspid regurgitation. Ejection Fraction was 50% and pulmonary artery systolic pressure was 50 mm Hg.- post thrombolytics: showed significant reduction in the peak and mean AV gradients back to baselines (66 and 41 mm Hg, respectively)	- Initial: stuck one of the AV leaflets in a closed & opening positions - post-thrombolytics: showed normal opening and closure of both discs	n/a	n/a	- Cardiac: stuck right (posterior) disc with a 5.9 mm × 4 mm (area 15.5 mm2) thrombus at the hinge point of fixed disc into the supra-valvular area, it has CT attenuation of 60 HU surrounded by pannus formation which is partially enhanced and CT attenuation of 194 HU - 48 h after thrombolytic therapy: reduction of the thrombus size to 2.7 × 2.4 mm	- 3 mo f/u transthoracic US: normal functioning mechanical AV

In these eight total cases incident PVT was associated with simultaneous or recent COVID-19 infection. All patients in these cases tested positive for COVID-19 within two months prior to the PVT diagnosis; three PVT cases were diagnosed during hospitalization with COVID-19 [7, 10, 12]. Additionally, five cases occurred greater than three years after the most recent valve replacement [5–8, 10], and the other three bioprosthetic valves failed within eight months of valve replacement [9, 11, 12]. One case, described by Vinnakota et al., documented a recurrent case of PVT: 12 months after initial mitral and aortic valve replacements, the patient underwent transcatheter mitral valve-in-valve implantation for suspected early mitral bioprosthetic stenosis secondary to PVT, and seven months afterwards was diagnosed with COVID-19, presenting with a second PVT within the next month.

Seven of eight cases document patients having been on anticoagulation and/or antiplatelet therapy leading up to the PVT, for anti-thrombotic purposes relating to prostheses, atrial fibrillation (AF), and/or recent COVID-19 infection. Acenocoumarol, warfarin, apixaban, or aspirin were used, and none had documented medication non-adherence noted [5–7, 9–12].

Of the six cases that included, inflammatory markers were noted to be increased when included in the case reports – including elevated D-dimer [5, 7, 8, 10–12], ranging from 372 ng/mL [10] to 6893 ng/mL [5].

Five of eight cases described severe COVID-19 infection including hospitalization with associated respiratory distress and associated abnormal lung findings seen on imaging [5–8, 11]; one case further developed measured pulmonary hypertension and respiratory failure [5], one developed pulmonary edema subsequent to supraventricular tachycardia [7] and one developed pulmonary congestion with pleural effusion [8]. In addition to PVT, two of the eight cases documented other thrombotic events also presumed to be associated with COVID-19 infection: Manghat et al. diagnosed pulmonary embolism, embolic myocardial infarction, and symptoms of retinal artery embolism, and Llopis Gisbert et al. also included pulmonary embolism. Of all six cases with recorded D-dimer levels, these two cases described by Manghat et al. and Llopis Gisbert et al. displayed the second (5547 ng/mL) and third (3041 ng/mL) highest levels, respectively.

Two of the eight total cases document a history of chronic disease prior to the COVID-19-associated PVT. Vinnakota et al. describe a patient with end-stage renal failure and AF that developed PVT with an imaged in situ thrombus measuring 20 × 15 × 20 mm. Cardona Buitrago et al. describe a patient with permanent valvular AF, obesity, congenital single kidney, and hypothyroidism that developed two PVT events within eight months (as stated earlier), the latter being associated with COVID-19 infection and described as a “large thrombus” resulting in “near total occlusion of flow.”

**Fig. 2:**
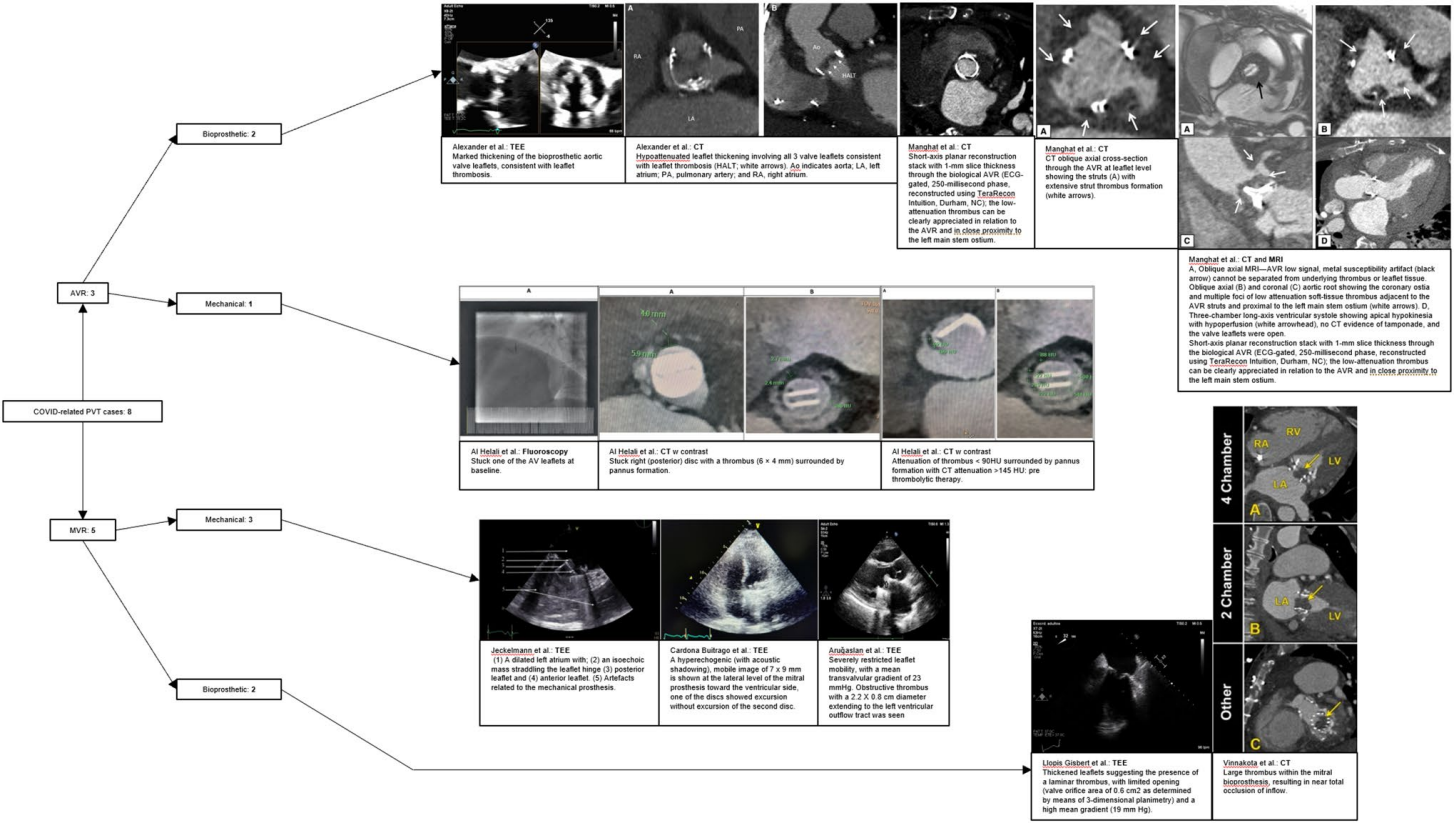
Collection of Images Presented with Selected Imaging Modalities. Top to Bottom: bioprosthetic aortic valve, mechanical aortic valve, mechanical mitral valve, bioprosthetic mitral valve. Left to Right Row 1 Alexander et al., TEE and CT images demonstrating leaflet thickening; Manghat et al., CT and MRI images visualizing paravalvular thrombus (arrows). Left to Right Row 2 Al Helali et al., Fluoroscopy and CT images demonstrating thrombus, pannus formation and restricted leaflet mobility. Left to Right Row 3 Jeckelman et al., TEE image demonstrating isoechoic mass; Cardona Buitrago et al., TEE image demonstrating hyperechogenic image; Aruğaslan et al., TEE image demonstrating restricted leaflet mobility and thrombus. Left to Right Row 4 Llopis Gisbert et al., TEE image demonstrating thrombus; Vinnakota et al., CT image demonstrating thrombus. TEE (Transesophageal Echocardiogram); CT (Computed Tomography); MRI (Magnetic Resonance Imaging) [5–12]

In all eight cases, the COVID-19-associated PVT was diagnosed with echocardiography, computed tomography, and/or fluorography. Results showed thrombotic mass attached to the prosthesis (if specified, to leaflet(s), disc, or disc hinge point), reduced mobility of prosthetic leaflet(s), and/or thickened leaflet(s) (Fig. 2).

Half of the eight cases documented additional complications/consequences – all cardiac related – aside from the COVID-19-assocated respiratory difficulties or thrombotic events already described above. In the case reported by Jeckelmann et al., the patient had developed a new onset AF specifically related to the PVT. In the case described by Manghat et al., the patient’s PVT and embolic MI were also accompanied by pericardial effusion (possibly perioperative after recent replacement) and transient atrial flutter. In the case described by Aruğaslan et al., the patient experienced supraventricular tachycardia and subsequent pulmonary edema as mentioned before, alongside the re-elevation of mean pressure gradient amid ineffective anticoagulation treatment; after thrombolytics proved ineffective, emergency valve replacement was done. Lastly, in the case described by Cardona Buitrago et al., during recovery after emergency valve replacement, the patient experienced significant bradyarrhythmia with rhythm of escape from the junction, occasional premature ventricular complexes of two morphologies, pairs, bigeminy, and episodes of non-sustained ventricular tachycardia; antiarrhythmics and a unicameral pacemaker had to be administered.

Regarding treatment for the COVID-19-associated PVT (and other thrombotic events), three patients were treated only with anticoagulation as their most intensive form of therapy, using heparin and warfarin [11, 12] or heparin alone [8]. Another three patients were treated with thrombolytics as their most intensive therapy, using alteplase [5, 10] or tenecteplase [9]. Finally, two cases, those described by Cardona Buitrago et al. and Aruğaslan et al., ultimately required emergency valve replacement. In both of these cases, the replaced valves were mechanical mitral valves implanted more than 3 years prior, COVID-19 infection was confirmed within 20 days prior to PVT, and the patients were on warfarin therapy leading up to the incident.

All eight patients survived to hospital discharge. Follow-up imaging studies in all seven of the cases that documented them, done soon after or months after these therapies, revealed resolution of PVT, normal prosthesis function, and/or improvement of valve gradient [5, 6, 8–12].

## Discussion

The hypercoagulable state associated with COVID-19 infection has been hypothesized to be related to host inflammatory response to the virus and associated increased inflammatory cytokine production [2–4]. Elbadawi et al. found that acute thrombotic events occurred in 5.2% of hospitalized patients with COVID-19; this 5.2% consisted of VTE (2.7%), ischemic stroke (1.2%), and MI (1.2%) [13]. Bilaoglu et al. reported an even higher incidence of thrombotic events among COVID-19 hospitalized patients (16.0%), with similar proportions [14]. Middeldorp et al. reported that of COVID-19-related VTE events, PE was the most frequent (81%) [15]. Based on our systematic review, the cases presented in this review are the only reported incidents of COVID-19-associated PVT. We support the authors’ proposed hypothesis that these eight incidents are at least partially a result of the pro-thrombotic nature of COVID-19 infection, as noted in this discussion.

It has been postulated that the severity of COVID-19 infection may correlate with the likelihood of subsequent thrombotic events, with the highest incidence among patients in the intensive care unit [16]. Consistent with these findings, most of these eight cases of PVT occurred in the context of severe COVID-19 infection, with patients being hospitalized for associated respiratory difficulties and displaying COVID-19 pneumonia lung lesions [5–8, 11].

The most correlative prognostic factor for severity/mortality of COVID-19 illness and associated thrombotic events is suggested to be elevated D-dimer [17–20]. Du et al. found a pooled odds ratio of 1.90 (95% CI: 1.32–2.48; P < 0.001) regarding the relationship between D-dimer and COVID-19, and Berger et al. found that patients with elevated D-dimer were more likely to have critical illness than those with normal D-dimer (43.9% versus 18.5%). Berger et al. also asserted that COVID-19 patients with elevated D-dimer were also more likely to undergo a thrombotic event (19.4% versus 10.2%). These findings are again consistent with the COVID-19-associated PVT cases presented in this review, with all six cases that included lab results displaying elevated D-dimer [5, 7, 8, 10–12]. Furthermore, Berger et al. found that patients with D-dimer > 2000 ng/mL had the highest risk of thrombotic event (37.8%), and it is interesting to note that four out of the six (66.7%) cases that included D-dimer levels in this review displayed values > 2000 ng/mL [5, 8, 11, 12]; perhaps an even higher D-dimer level–- and a more hypercoagulable state–- is associated with PVT compared to other thrombotic events associated with COVID-19 infection.

The general risk factors of PVT have been well described. Dürrleman et al. report that the mean time interval from first valve replacement to PVT was 39 ± 42 months [21], and it has been documented that PVT incidence is highest during the early post-operative period for both mechanical and bioprosthetic valves [22]. In comparison, PVT ranged from 2 weeks to 22–24 years) from first valve replacement in COVID-19-associated PVT for the eight cases in this review; five of these cases occurred greater than 3 years post-replacement [5–8, 10]. This wide range of time until PVT suggests COVID-19 infection might increase the risk of late PVT.

In general, mechanical valve PVT is generally more prevalent compared with bioprosthetic valve PVT [22, 23], with thrombosis incidence rate being 3–190 times greater for mechanical valves (0.1–5.7%) than for bioprosthetic valves (0.03%) [23]. For the eight cases in this review, the number of PVT incidents involving mechanical valves vs bioprosthetic valves were equal. This offers a possible hypothesis that COVID-19 infection may be associated with increased PVT risk in bioprosthetic valves. Mitral valve PVT is considered 2–3 times more frequent than aortic PVT [22], with 67% of all PVTs involving the mitral position and 15% involving the aortic position [21]. A roughly similar proportion was found in this review, with 5/8 (62.5%) involving mitral PVT and 3/8 (37.5%) involving aortic PVT; offering a hypothesis that COVID-19 infection may be associated with PVT independent of mitral or aortic valve position.

The most frequent risk factors contributing to PVT include inadequate anticoagulation, AF, recent infection, and plasma fibrinogen level, as reported by Bezanjani et al. Furthermore, with regards to inadequate anticoagulation, it has been recently recognized that public policy-related COVID-19 restriction measures have posed a challenge for patients and physicians to regularly assess patients’ INR levels and ensure effective anticoagulation; Vriz et al. found that the incidence of stuck prosthetic valves has significantly increased amid these restrictions [24]. While we acknowledge that COVID-19 restriction measures themselves may have had an influence on PVT incidence during the COVID-19 pandemic, it is important to note that none of the cases included in this review noted significant non-adherence to anticoagulation medication nor INR check-ups. This suggests that COVID-19 infection itself may increase the risk of PVT. In the context of the reported PVT cases presented, COVID-19 infection potentiated PVT and overcame the therapeutic effect of anticoagulation in those patients taking it. Thus, patients with prosthetic valves –both mechanical and bioprosthetic – and recently diagnosed with COVID-19 infection may warrant closer monitoring or more frequent therapeutic monitoring, and possibly higher doses of anticoagulants to potentially lower the risk of PVT. PVT is an important topic for future and ongoing investigation in better characterizing risks and risk groups of patients infected with COVID-19.

The patients included present heterogeneously and seemingly unpredictably. Limitations of this review include the inclusion criteria of English only publications and the inherent newness of COVID-19 and the evolving understanding of the various manifestations of interactions it may pose both short term and long term on human physiology and specifically valve prostheses. As COVID-19 continues to evolve and dynamically change and global prevalence continues, infection in the growing cohort of prosthetic valve patients with waning natural and vaccine related immunity or absent immune protection from emerging COVID-19 variants may further demonstrate clinical relevance of PVT in the setting of COVID-19 infection. Early diagnosis of PVT can modify treatment recommendations and improve the quality of care in patients with prosthetic valves.

## Conclusions

With the known hypercoagulability associated with COVID-19 infection, prosthetic valve failure and valve thrombosis is an important diagnosis to consider in hospitalized COVID-19 infected patients.

## Clinical implications

Prosthetic valve failure of the aortic and mitral valve positions thrombosis has been reported in patients with both mechanical and tissue valve prostheses with or without ongoing antithrombotic or antiplatelet therapy in the setting of COVID-19 infection. In patients with prosthetic valves, COVID-19 related valve failure is plausible to include as a consequence of infection.

## Data Availability

Not applicable.

## Abbreviations

PVT: Prosthetic Valve Thrombosis
COVID: SARS-CoV-2
